# SVMMDR: Prediction of miRNAs-drug resistance using support vector machines based on heterogeneous network

**DOI:** 10.3389/fonc.2022.987609

**Published:** 2022-09-29

**Authors:** Tao Duan, Zhufang Kuang, Lei Deng

**Affiliations:** School of Computer and Information Engineering, Central South University of Forestry and Technology, Changsha, China

**Keywords:** miRNA, drug resistance, support vector machines, hetesim score, random walk with restart

## Abstract

In recent years, the miRNA is considered as a potential high-value therapeutic target because of its complex and delicate mechanism of gene regulation. The abnormal expression of miRNA can cause drug resistance, affecting the therapeutic effect of the disease. Revealing the associations between miRNAs-drug resistance can help in the design of effective drugs or possible drug combinations. However, current conventional experiments for identification of miRNAs-drug resistance are time-consuming and high-cost. Therefore, it’s of pretty realistic value to develop an accurate and efficient computational method to predicting miRNAs-drug resistance. In this paper, a method based on the Support Vector Machines (SVM) to predict the association between MiRNA and Drug Resistance (SVMMDR) is proposed. The SVMMDR integrates miRNAs-drug resistance association, miRNAs sequence similarity, drug chemical structure similarity and other similarities, extracts path-based Hetesim features, and obtains inclined diffusion feature through restart random walk. By combining the multiple feature, the prediction score between miRNAs and drug resistance is obtained based on the SVM. The innovation of the SVMMDR is that the inclined diffusion feature is obtained by inclined restart random walk, the node information and path information in heterogeneous network are integrated, and the SVM is used to predict potential miRNAs-drug resistance associations. The average AUC of SVMMDR obtained is 0.978 in 10-fold cross-validation.

## 1 Introduction

In recent years, the difficulty of drug target selection has led to the increase of drug development cost and the low efficiency of pharmaceutical industry. So far, it has been discovered that the human genome can encode up to 25,000 genes. But only 600 of the disease-causing proteins have targeted drugs, meaning a significant number are “undrugable”. Therefore, the focus of target selection has now shifted to other macromolecules, such as non-coding RNA.

According to the genetic central dogma, the DNA is a carrier that carries genetic information. During growth and development, the genetic information in DNA is transcribed into RNA. Then the RNA is translated into various proteins to perform specific biological functions. There are many types of RNAs with complex functions. Research shows that only 2% of the RNA could code for proteins, and 98% couldn’t. In biology, RNAs with non-coding are called non-coding RNAs (ncRNAs). Among ncRNAs, miRNA is a group of non-coding Rnas encoded by the genome with a length of about 20̴23 nucleotides. The miRNAs play an major role in gene expression regulation. They have a significant meaning in many biological processes such as cell differentiation, development and cellular signaling pathways.

The miRNAs play an important role in the understanding of life sciences. The miRNAs are significant in many aspects such as cellular biological processes, gene expression regulation at transcriptional and post-transcriptional levels, and others.

There are many studies on the biological mechanisms and interactions between genes, miRNAs, lncRNAs, diseases and drugs, such as the relationship between genes and diseases, miRNAs and diseases, lncRNAs and diseases, miRNAs and lncRNAs, etc.

For the association between genes and diseases, a network impulse dynamics framework based on multiple biological networks NIDM is proposed by Xiang et al. (1) to predict potential disease-gene associations. The HyMM is proposed by Xiang et al. (2) to more effectively predict disease-related genes by integrating information from the structure of multi-scale modules. The PrGeFNE is proposed by Xiang et al. (3) based on fast network embedding. An understanding of the association between genetics and disease can help understand the pathogenesis of disease.

For the association between miRNAs and diseases, a meta-path-based MDPBMP is proposed by Yu et al. (4). The information carried by the nodes is extracted and integrated through MDPBMP, and the miRNAs-disease association is predicted using embedded feature vectors. The VGAE-MDA, a deep learning framework with variational graph autoencoder, is proposed by Ding et al. (5). The MLPMDA, a miRNAs-disease association prediction method using multilayer linear projection, is proposed by Guo et al. (6). The prediction method GRPAMDA is proposed by Zhong et al. (7). The GRPAMDA combines the graph random propagation network based on DropFeature and attention network. The NIMGSA is proposed by Jin et al. (8) to predict miRNAs-disease association based on neural induction matrix completion. An ensemble learning framework with resampling method for miRNA-disease association ERMDA prediction is proposed by Dai et al. (9). A double random walk model is proposed by Zhu et al. (10). The end-to-end deep learning method PDMDA is proposed by Yan et al. (11). A computational framework SMALF based on XGBoost is proposed by Liu et al. (12). The algorithm MSCDE is proposed by Han et al. (13) based on a variety of biological source information. The method based on tensor factorization and label propagation (TFLP) is proposed by Yu et al. (14) for multi-type miRNA-disease association prediction.

For the association between lncRNAs and diseases, a non-negative matrix factorization based on graph regularization LDGRNMF is proposed by Wang et al. (15) to predict the lncRNAs-disease association. The internal inclined random walk with restart (IIRWR) is used by Wang et al. (16) to infer potential lncRNA-disease associations. A lncRNAs-disease association prediction method GBDTLRL2D based on Gradient Boosting Decision Tree and Logistic Regression is proposed by Duan et al. (17). The GCRFLDA, a prediction method based on graph convolution matrix completion, is proposed by Fan et al. (18). An end-to-end computational method based on graph attention network GANLDA is proposed by Lan et al. (19). A method called LRWRHLDA is proposed by Wang et al. (20). The LRWRHLDA designs a multi-layer network using six known heterogeneous networks, and uses Laplace normalized random walk and restart algorithm to predict. A dual attention network is proposed by Liu et al. (21).

For the association between miRNAs and lncRNAs, the LMI-INGI, based on interactome network and graphlet interaction, is proposed by Zhang et al. (22) to predict the lncRNAs-miRNAs associations. The NALMA is proposed by Zhang et al. (23) to use network distance analysis. The DWLMI proposed by Yang et al. (24). utilizes lncRNAs-miRNAs-disease-protein-drug diagram. The structural perturbation method SPMLMI is proposed by Xu et al. (25). A logical matrix factorization with neighborhood regularized, LMFNRLMI, is proposed by Liu et al. (26).

Advances in genomics and bioinformatics have facilitated the identification of miRNAs. The miRNAs have also been found to interact with a variety of drugs. It is possible to develop resistance or sensitivity during drug treatment because of the regulation of genes by miRNAs (27). For example, scientists have found that miRNA let-7b is resistant to the drug cisplatin (28). Cisplatin is an important drug in the treatment of many diseases, such as sarcoma. Cisplatin has also been reported to down-regulate miRNA let-7b expression, lead to up-regulation of Cyclin D1, and induce resistance to cisplatin. Similarly, miRNA Mir-106a is found to enhance the sensitivity of OVCAR3/CIS cells to cisplatin (29). Since both the increase and decrease of miRNA expression level can cause diseases, miRNA-targeted therapy drugs can be divided into miRNA mimics and miRNA inhibitors. Their aim is to induce gene silencing and selective up-regulation of proteins.

There are several public databases that collate miRNAs-drug relationships. For instance, the database of miRNAs-drug interactions, pharmaco-miR is developed by Rukov et al. (30) according to the interaction between miRNA target genes and drug proteins. The database mTD is developed by Chen et al. (31) to collect information about the impact of miRNAs during drug treatment. The ncDR is developed by Dai et al. (32) to provide information of noncoding RNAs related to drug resistance. However, the known link between miRNAs and drug resistance is limited. Because biological experiments are time-consuming and expensive, it is necessary to develop computation-based methods to predict the potential association between miRNAs and drug resistance.

Different computational methods have been developed to identify and predict miRNAs-drug resistance. For example, an algorithm for predicting potential miRNAs-drug resistance associations through Bi-Random Walk (BiRW) is proposed by Xu et al. (33). The method SNMFSMMA based on symmetric non-negative matrix factorization and kronecker regularized least squares is developed by Zhao et al. (34) for prediction of small molecular-miRNAs association. The GCMDR proposed by Huang et al. (35) uses graph convolution to built potential factor model, learns the graph embedding feature of miRNAs and drugs, and expresses the problem of predicting miRNA-drug association as a link prediction problem involving two- miRNA-drug sensitivity associations, named LGCMDS, is proposed by Yu et al. (36). The MDIPA, a matrix factor-based method, is proposed by Jamali et al. (37) to predict the unknown interactions between miRNAs and drug resistance. Predicting associations between small molecular and microRNAs using functional similarity of miRNAs and multiple similarity measures of small molecular is proposed by Qu et al. (38). In addition, combined with clinical, chemical, and biological information, a method based on non-negative matrix factorization to predict miRNAs-small molecule relationships is developed by Luo et al. (39).

Although there are some studies on predictive tools for miRNAs-drug resistance associations, these methods cannot fully utilize the structure and semantics in heterogeneous networks to extract higher-quality information. At the same time, the accuracy and performance obtained by these methods need to be improved. To address these issue, a method for predicting miRNAs-drug resistance based on support vector machines SVMMDR is proposed in this paper. The SVMMDR considers the path information between nodes in heterogeneous networks. The hetesim measures the correlation between nodes of the same type or different types within a unified framework. At the same time, based on the search path between two nodes, the measure between node pairs is defined by following a sequence. The node information and path information in heterogeneous networks are integrated. The SVM is used to predict potential miRNAs-drug resistance associations. The contribution of our method mainly consists of the following parts:

- The SVMMDR introduces the concept of miRNA and drug groups. On this basis, a roaming network is established. Walker is more inclined to choose the next node of the walk. The inclined diffusion feature is obtained by inclined restart random walk.
- The SVMMDR integrates node information and path information in heterogeneous networks. The data feature is obtained by combining the inclined diffusion feature and hetesim score.
- The SVMMDR algorithm improves prediction accuracy and has the highest AUC values when compared to existing algorithms.

## 2 Materials and methods

The miRNAs-drug resistance association data required in this paper are downloaded from ncDR database (32). The ncDR collected 5864 validated relationships between 145 compounds and 1039 ncRNAs (877 miRNAs and 162 lncRNAs) from approximately 900 published papers. We only need the correlation between miRNAs-drug resistance among them. After removing duplicate data, the 625 miRNAs, 85 drugs and 2301 miRNAs-drug resistance associations are obtained.

**Figure 1:**
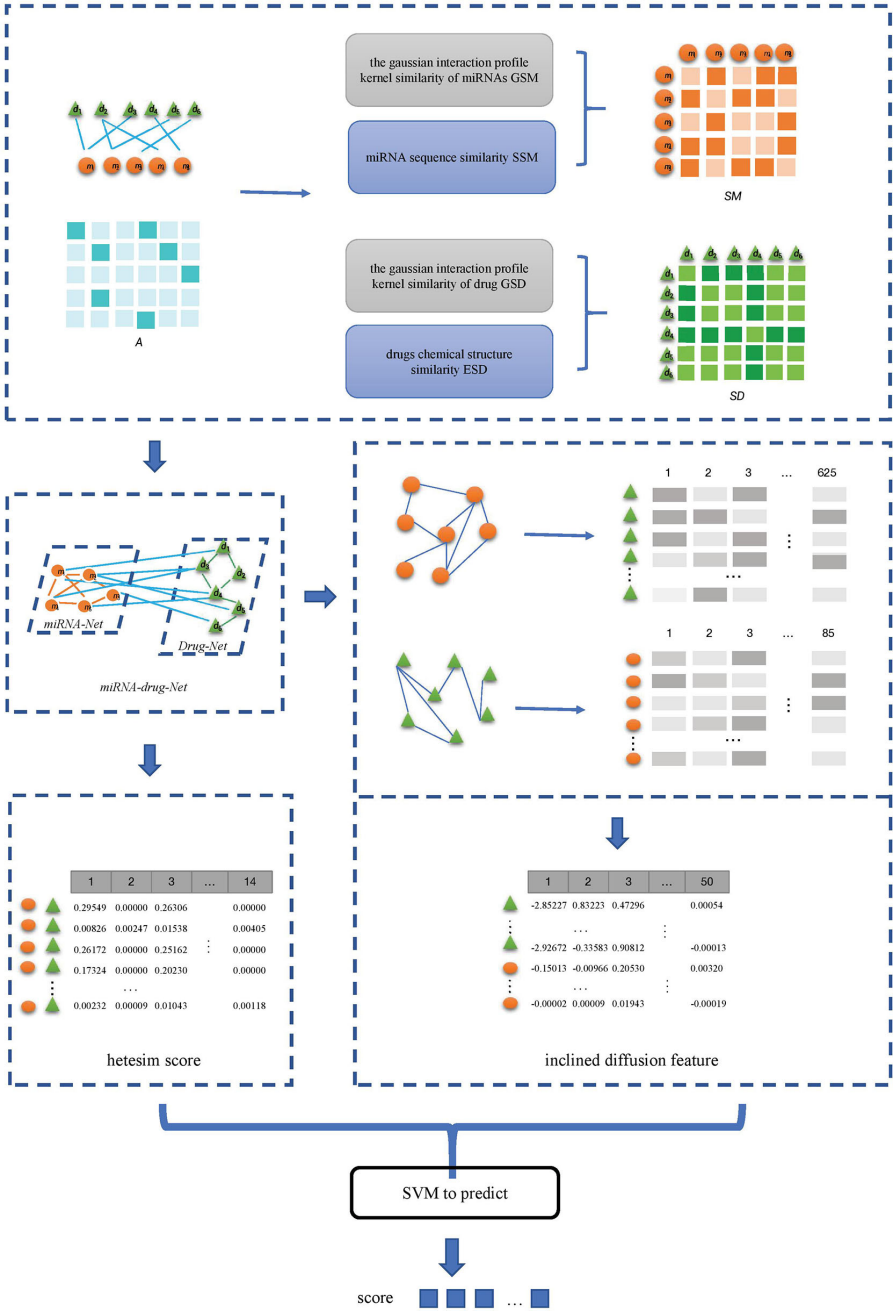
Flowchart of the SVMMDR.

In this paper, an SVM-based method SVMMDR is proposed to predict the association between miRNAs-drug resistance. The SVMMDR integrates miRNAs-drug resistance association, miRNAs sequence similarity, drugs chemical structure similarity and other similarities. The path-based hetesim feature is obtained, and the concepts of miRNAs group and drug group are introduced to obtain the inclined diffusion feature through inclined random walk. Finally, the SVM algorithm is used to predict the association between miRNAs and drug resistance. This mainly includes the following steps:

(1) The miRNAs-drug resistance association data set is downloaded from the ncDR, and the list of miRNAs and drugs, the matrix A of miRNAs-drug resistance association are obtained by de-duplication of the downloaded data. Then the gaussian interaction profile kernel similarity matrix of miRNAs GSM and of drug GSD are calculated.
(2) The sequence of miRNA list is downloaded from miRBase database, and the miRNAs sequence similarity matrix SSM between miRNAs is calculated. The drug chemical structure similarity matrix ESD is obtained by using the published tool SimComp.
(3) The miRNAs similarity network is obtained based on the GSM and SSM, and drugs similarity network is obtained based on the GSD and ESD.
(4) The miRNA-resistance association network, miRNA similarity network, and drug similarity network are integrated to construct a heterogeneous network. In the heterogeneous networks, the inclined diffusion feature are obtained based on the inclined random walk with restart. Then the low-dimensional inclined diffusion feature are obtained by using Singular Value Decomposition (SVD).
(5) The hetesim scores for miRNA-drug pairs are calculated based on paths in the heterogeneous network.
(5) The inclined diffusion feature and the hetesim score are combined to obtain the feature data set. The combined features are used in the SVM classifier to obtain the predicted scores for miRNAs-drug resistance. The flow of SVMMDR is shown in **Figure 1**.

### 2.1 Calculate Gaussian interaction profile kernel similarity

The matrix A of miRNAs-drug resistance association network is obtained. The number of rows of A is the number of miRNAs, and the number of columns of A is the number of drugs, as shown in the formula (1):


(1) $$A(m_{i},d_{j})=\{\begin{array}{cc} 1 & m_{i}\text{ is associated with }d_{j}\\ 0 & \text{ otherwise } \end{array}$$

Where *A*(*m_i_*, *d_i_*) = 1 indicates that there is a resistance between miRNA *m_i_* and drug *d_j_*.

For any given miRNA *m_i_* and *m_j_*, the gaussian interaction profile kernel similarity GSM(*m_i_*, *m_j_*) can be obtained based on A, as shown in the formula (2) and (3):


(2) $$\text{GSM(}m_{i},m_{j})=\text{exp }\left(-\delta_{m}{ǁA(i,:)-A(j,:)ǁ}^{2}\right)$$

(3) $$\delta_{m}=\delta_{m}^{'}/\left(\frac{1}{nm}\sum_{i=1}^{nm}ǁ\text{A}{(i,:)ǁ}^{2}\right)$$

where *nm* is the number of miRNAs and *A*(*i*, ): is the *i_th_* row of the adjacency matrix *A*. The δ*_m_* is used to control the frequency band, it represents the normalized frequency band of Gaussian interaction profile kernel similarity based on the new frequency band parameter δ’*_m_*. The gaussian interaction profile kernel similarity between drugs can be obtained in the same way, represented by GSD, which is given by (4) and (5):


(4) $$\text{GSD(}d_{x},d_{y})=\text{exp }\left(-\delta_{d}{ǁA(:,x)-A(:,y)ǁ}^{2}\right)$$

(5) $$\delta_{d}=\delta_{d}^{'}/\left(\frac{1}{nd}\sum_{x=1}^{nd}{ǁA\left(:,x\right)ǁ}^{2}\right)$$

where *nd* is the number of miRNAs and *A*(: *x*) is the *x_th_* col of the *A*.

### 2.2 Calculate miRNA sequence similarity and drug chemical structure similarity

The sequences of relevant miRNAs are downloaded from the public database miRBase (https://mirbase.org/) (40). The miRBase database provides information including miRNAs sequence data, annotations, and predicted gene targets. The sequence similarity SSM between miRNAs is calculated as shown in the formula (6):


(6) $$\text{SSM (}m_{i},m_{j})=1-\frac{\;Levenshtein(m_{i},m_{j})}{len(m_{i})+len(m_{j})}$$

(7) $$0\leq \text{ Levenshtein }(m_{i},m_{j})\leq \text{len (}m_{i})+\text{len(}m_{j})$$

Where len(*m_i_*) represents the length of miRNA *m_i_* sequence, len(*m_j_*) represents the length of miRNA *m_j_* sequence, Levenshethein(*m_i_*, *m_j_*) is defined as the class editing distance of the transformation from *m_i_* sequence to *m_j_* sequence.

With the Kyoto Encyclopedia of Genes and Genomes (KEGG) database entry number corresponding to drugs in the DLREFD database as the parameter, the chemical structural similarity matrix ESD between drugs is calculated using the SimComp tool (41).

### 2.3 Integer similarity

In this section, miRNAs similarity network and drugs similarity network are constructed. The miRNAs similarity network is expressed as SM. The SM is fused by SSM and GSM, which is given by (8):


(8) $$\text{SM (}m_{i},m_{j})=\{\begin{array}{ll} GSM(m_{i},m_{j}) & \text{ if }SSM(m_{i},m_{j})=0\\ SSM(m_{i},m_{j}) & \text{ otherwise } \end{array}$$

Similarly, denote SD as the drug similarity network, which is fused by ESD and GSD, as follows:


(9) $$\text{SD (}d_{i},d_{j})=\{\begin{array}{ll} GSD(d_{i},d_{j}) & \text{ if }SSM(d_{i},d_{j})=0\\ SSD(d_{i},d_{j}) & \text{ otherwise } \end{array}$$

### 2.4 Obtain low-dimensional network inclined diffusion features

A global heterogeneous network is constructed by integrating the association matrix A of miRNAs-drug resistance network, the similarity matrix SM of miRNA and the similarity matrix SD of drugs. The concepts of miRNAs group and drugs group are introduced to obtain miRNA weight matrix and drug weight matrix to construct roaming network. The restart random walk is used to calculate the inclined diffusion feature on the roaming network, and the high dimensional inclined diffusion feature are obtained. Then, the SVD is used to reduce the dimension of the high-dimensional inclined diffusion feature, and the low-dimensional inclined diffusion feature is obtained. The specific sub-steps are as follows:

#### 2.4.1 Building a heterogeneous network

The heterogeneous network G = (*V*, *E*) is constructed. The dimension of the matrix G is (*nm* + *nd*) * (*nm* + *nd*), where *nm* and *nd* is the number of miRNAs and drugs, as shown in formula (10):


(10) $$\text{G}=\left[\begin{array}{cc} SM & A\\ A^{T} & SD \end{array}\right]$$

where *A* ^T^ is the transpose of A.

#### 2.4.2 Obtain the weight matrix

The drugs associated with the same miRNA are regarded as a drug group. If one miRNA with high similarity to this miRNA are associated with a drug in this drug group, this miRNA is considered to have a potential association with other drugs in the drug group.

For example, for drug *d_i_* , miRNAs associated with *d_i_* are regarded as a miRNA group. If *d_j_* with high similarity to *d_i_* is associated with miRNA in this miRNA group, then it is assumed that *d_i_* may be associated with other miRNAs in the miRNA group. Based on the above assumptions, miRNAs weight matrix *W_MM_* of *nd* * *nm* dimension and drugs weight matrix *W_DD_* of *nm* * *nd* dimension are obtained, as shown in the formula (11) and (12):


(11) $$W_{MM}(d_{i},m_{j})=\frac{SS_{M}(d_{i},m_{j})}{\underset{1\leq i\leq nd}{\max}\left\{SS_{M}(d_{i},m_{j})\right\}}$$

(12) $$SS_{M}(d_{i},m_{j})=\sum_{m_{k}\in DM(d_{i})}SM(m_{k},m_{j})$$

where *DM* (*d_i_*) = {*m_k_* | ∀*m_k_* ∈ {*if*(*A* (*m_k_*, *d_i_*) = = 1)},1 ≤ k ≤ nm} represents the miRNA group associated with the drug *d_i_*. *if* (*A* (*m_k_*, *d_i_*) = = 1, 1 ≤ *k* ≤ *nm*} represents that miRNA *m_k_* is associated with drug *d_i_*. *SM*(*m_k_*, *m_j_*) is the similarity between miRNA *m_k_* and *m_j_*.

The drugs weight matrix *W_DD_* of *nm* * *nd* dimension can also be obtained:


(13) $$W_{DD}(m_{j},d_{i})=\frac{SS_{D}(m_{j},d_{i})}{\underset{1\leq j\leq nm}{\max}\left\{SS_{D}(m_{j},d_{i})\right\}}$$

(14) $$SS_{D}(m_{j},d_{i})=\sum_{d_{z}\in DD(m_{j})}SD(d_{z},d_{i})$$

where *DD* (*m_j_*) = {*d_z_* | ∀*d_z_* ∈ {*if* (*A*(*d_z_, m_j_*) = = 1)}, 1 ≤ *z* ≤ *nd*} represents the drug group associated with the drug *m_j_*. *if* (*A*(*d_z_, m_j_*) = = 1)}, 1 ≤ *z* ≤ *nd*} represents that drug *d_z_* is associated with drug *m_j_*. *SM* (*d_z_*, *m*_j_) is the similarity between drug *d_z_* and *m_j_*.

#### 2.4.3 Construct roaming network

When drug *d_x_* is a walker, it walks on the miRNAs node network. *T_D_* is the transition probability matrix of the roaming network. For any given miRNA *m_i_* and *m_j_*, denote *T_D_* as the probability of *m_i_* transferring to *m_i_* during the walking process.


(15) $$T_{D}(m_{i},m_{j})=\frac{ST_{D}(m_{i},m_{j})}{\sum_{n=1}^{nm}ST_{D}(m_{n},m_{j})}$$

(16) $$ST_{D}(m_{i},m_{j})=\{\begin{array}{ll} W_{DD}(m_{j},d_{x}) & \text{ If }W_{DD}(m_{j},d_{x})>0\\ SM(m_{i},m_{j}) & \text{ otherwise } \end{array}$$

where, *m_i_* is the current node of migration, and *m_j_* is the next node. If the value of *m_j_* and *d_x_* in the weight matrix is not 0, it means that *m_j_* and *d_x_* have potential correlation, namely *ST_D_* (*m_i_*, *m_j_*) = *W_DD_* (*m_j_*, *d_x_*). Otherwise, the probability of *m_i_* transferring to *m_j_* is related to miRNA similar matrix, *ST_D_* (*m_i_*, *m_j_*) = *SM* (*m_i_*, *m_j_*).

When miRNA *m_y_* is a walker, it walks on the miRNAs node network. Denote *T_M_* as the transition probability matrix of the roaming network. For any given drug *d_i_* and *d_i_*, *T_M_* represents the probability of *d_i_* transferring to *d_i_* during the walking process.


(17) $$T_{M}(d_{i},d_{j})=\frac{ST_{M}(d_{i},d_{j})}{\sum_{n=1}^{nd}ST_{M}(d_{n},d_{j})}$$

(18) $$\begin{array}{c} ST_{M}(d_{i},d_{j})=\{\begin{array}{ll} W_{MM}(d_{j},d_{x}) & \text{ If }W_{MM}(d_{j},m_{y})>0\\ SD(d_{i},d_{j}) & \text{ otherwise } \end{array} \end{array}$$

#### 2.4.4 Obtain inclined diffusion feature by IIRWR

Based on the transfer probability matrix *T_D_* and *T_M_* obtained, the drugs inclined diffusion feature *P_D_* = [*P* ^1^, *P* ^2^, *P* ^3^,…, *P^x^*,…,*P^nd^*] can be obtained by random walk, where *P^x^* represents the *nm*-dimensional inclined diffusion feature of drug node *d_x_*. Meanwhile, the miRNAs inclined diffusion feature *P_M_* = [*P* ^1^, *P* ^2^, *P* ^3^,…, *P^y^*,…,*P^nm^*], where *P^y^* denotes the *nd*-dimensional inclined diffusion feature of miRNA node *m_y_*. The *nm* and *nd* denote the number of miRNA nodes and drug nodes.

When the inclined diffusion feature *P^x^* of drug node *d_x_* is calculated, each step of the walking is faced with two choices: randomly selecting adjacent miRNA node or returning to the starting node. The walking process is shown in the equation (19):


(19) $$P_{t+1}^{x}=(1-r)\times T_{D}\times P_{t}^{x}+r\times P_{0}^{x}$$

(20) $$P_{0}^{x}(m_{i})=\frac{A(m_{i},d_{x})}{\sum_{n=1}^{nm}A(m_{n},d_{x})}$$

When the inclined diffusion feature *P^y^* of miRNA node *m_y_* is calculated, the walking process is shown as follows:


(21) $$P_{t+1}^{y}=(1-r)\times T_{M}\times P_{t}^{y}+r\times P_{0}^{y}$$

(22) $$P_{0}^{y}(d_{j})=\frac{A(m_{y},d_{j})}{\sum_{n=1}^{nd}A(m_{y},d_{j})}$$

Where *r* is the restart probability, $P_{t}^{y}$ is a *nd*-dimensional transition probability vector of node *m_y_*, and its k-th element represents the probability of accessing node k at t step, *k* ∈ {1, 2, … *nd*}. $P_{0}^{y}$ represents the initial migration probability vector of node *m_y_*, and $P_{0}^{y}(d_{j})$ represents the initial migration probability of *m_y_* visiting node *d_j_*.

After several iterations, the difference between the two iterations of *p^x^* and *p^y^* is less than 10^-10^. The miRNA inclined diffusion feature *P_M_* and the drug inclined diffusion feature *P_D_* reach a stable state, and the final inclined diffusion feature is obtained.

#### 2.4.5 Calculate the low-dimensional inclined diffusion feature

The more nodes in the heterogeneous network, the higher the feature dimension obtained by the inclined restart random walk. However, when the feature dimension is high, there will be data redundancy. The sample distribution of the high-dimension space is sparse. The SVD is used to reduce the dimension of the inclined diffusion feature.

Suppose that the m * n dimensional matrix P can be decomposed by *P* = *U*Σ*V^T^*, where *U* is a m * m-dimensional matrix and *V* is a n * n-dimensional matrix. The *U* and *V* are left singular vectors and the right singular vectors, both unitary matrices, that is, *UU^T^ = 1*, *VV^T^ = 1*. The m * n dimensional matrix Σ has values only on the main diagonal, and all other elements are zero. Every element along the main diagonal is called singular value. The singular values are arranged from largest to smallest, and the decrease is extremely fast. In many cases, the sum of the first 10% or even 1% of the singular values accounts for more than 99% of the total singular values. In other words, we can also use the largest *d* singular values and corresponding left and right singular vectors to approximate the matrix, as follows:


(23) $$P_{m*n}=U_{m*m}{\text{Σ}}_{m*n}V_{n*n}^{T}\approx U_{m*d}{\text{Σ}}_{d*d}V_{d*n}^{T}$$

where *d* is far less than *n*, and the low-dimensional feature vector *X* can be obtained by formula (24):


(24) $$X=U_{m*d}{\left({\text{Σ}}_{d*d}\right)}^{1/2}$$

The SVD is performed on *P_D_* and *P_M_* respectively to obtain low-dimensional node feature matrix *X_D_* and *X_M_*.

### 2.5 Calculate the hetesim score

In heterogeneous networks, the types of nodes are different, and the relationship between nodes has various meanings. In order to obtain the correlation between different nodes, the hetesim scores are calculated (42). The hetesim is a path-based measurement method used to measure the correlation of objects (including objects of the same type or different types) in heterogeneous networks.

(1) The transition probability matrix I_MD f_rom miRNA to drug, I_DD f_rom drug to drug, I_MM f_rom miRNA to miRNA are obtained as follows:


(25) $${\text{I}}_{\text{MD}}(m_{i},d_{j})=\frac{A(m_{i},d_{j})}{\sum_{k=1}^{nd}A(m_{i},d_{k})}$$

(26) $${\text{I}}_{\text{DD}}(d_{i},d_{j})=\frac{SD(d_{i},d_{j})}{\sum_{k=1}^{nd}SD(d_{i},d_{k})}$$

(27) $${\text{I}}_{\text{MM}}(m_{i},m_{j})=\frac{SM(m_{i},m_{j})}{\sum_{k=1}^{nm}SM(m_{i},m_{k})}$$

**(2)** The N is the node, and there are only miRNA and drug node. The path with length *l* between any two nodes is represented by ρ=N_1_ N_2_⋯N*_l_*_+1_, and the reachable probability matrix PM=I_N_1_N_2__I_N_2_N_3__⋯I_N_*l*_N_*l*+1__ . Divide the path in half, get the *PM*_*ρ*_*L*__ and *PM*_*ρ*_*R*__.

- when *l* is even, $\rho_{L}={\text{N}}_{1}{\text{N}}_{2}\cdots {\text{N}}_{\frac{l}{2}+1}$ . $\rho_{R}={\text{N}}_{\frac{l}{2}+1}{\text{N}}_{\frac{l}{2}+2}\cdots {\text{N}}_{l+1}$ . The PM_ρL_ and PM_ρR_ is calculated.
- When *l* is odd, $\rho_{L_{1}}={\text{N}}_{1}{\text{N}}_{2}\cdots {\text{N}}_{\frac{l+1}{2}}$ , $\rho_{L_{2}}={\text{N}}_{1}{\text{N}}_{2}\cdots {\text{N}}_{\frac{l+3}{2}}$ . $\rho_{R_{1}}={\text{N}}_{\frac{l+1}{2}}{\text{N}}_{\frac{l+1}{2}+1}\cdots {\text{N}}_{l+1}$ . $\rho_{R_{2}}={\text{N}}_{\frac{l+3}{2}}{\text{N}}_{\frac{l+3}{2}+1}\cdots {\text{N}}_{l+1}$ . Then $\text{PM}\rho_{L}=\frac{PM_{\rho_{L_{1}}}+PM_{\rho_{L_{2}}}}{2}$ , $\text{PM}\rho_{R}=\frac{PM_{\overset{\circ }{h}o_{R_{1}}}+PM_{\rho_{R_{2}}}}{2}$ .

**(3)** The *PM*_*ρ*_*L*__ and ${\text{PM}}_{\rho_{R}^{-1}}$ are calculated, where $\rho_{R}^{-1}$ represents the reverse of *ρ*_R_, for example, if *ρ*_R_ = MMMDD, the $\rho_{R}^{-1}=\text{DDMMM}$ .


(28) $$Hetesim(a,bǀ\rho )=\frac{PM_{\rho_{L}}{\left(PM_{\rho_{R}^{-1}}\right)}^{T}}{ǀǀPM_{\rho_{L}}ǀ{ǀ}_{2}*ǀǀPM_{\rho_{R}^{-1}}ǀ{ǀ}_{2}}$$

where *Hetesim* (*a*, *b*| *ρ*) represents the hetesim score of the node *a* reaching the node *b* through path *ρ*. As shown in **Table 1**, there are 14 different paths from a miRNA to a drug when the *l*< 5. So, the 14-dimensional hetesim feature between each miRNA-drug node pair in the heterogeneous network is obtained.

**Table 1:** The paths from a miRNA to a drug in the heterogeneous network when *l*< 5.

id	path	meaning
1	MMD	miRNA-miRNA-drug
2	MDD	miRNA-drug-drug
3	MMMD	miRNA-miRNA-miRNA-drug
4	MDMD	miRNA-drug-miRNA-drug
5	MMDD	miRNA-miRNA-drug-drug
6	MDDD	miRNA- drug-drug-drug
7	MMMMD	miRNA-miRNA-miRNA-miRNA-drug
8	MMMDD	miRNA-miRNA-miRNA-drug-drug
9	MMDMD	miRNA-miRNA-drug-miRNA-drug
10	MMDDD	miRNA-miRNA-drug-drug-drug
11	MDMMD	miRNA-drug-miRNA-miRNA-drug
12	MDMDM	miRNA-drug-miRNA- drug-drugdrug
13	MDDMD	miRNA-drug-drug-miRNA-drug
14	MDDDD	miRNA-drug-drug-drug-drug

### 2.6 Training the support vector machine classifier

Feature data are obtained by combining inclined diffusion feature and hetesim score. For each pair of drug and miRNA sample in the calculated Hetesim score matrix, the 50-dimensional inclined diffusion feature of the corresponding miRNA and drug are obtained respectively, and 114-dimensional feature is obtained. For example, sample drug *d_i_* and miRNA *m_j,_* the 14-dimensional HeteSim score of *d_i_* – *m_j_* pair is combined with the 50-dimensional inclined diffusion feature of the corresponding drug *d_i_* and the 50-dimensional inclined diffusion feature of miRNA *m_j_*, namely, the i-th row of the *X_D_* and the j-th row of the matrix *X_M_*, to obtain the 114-dimensional feature of drug *d_i_* and miRNA *m_j_*. The 114-dimension feature dataof all sample pair are obtained by a similar method. The obtained feature data are used for SVM classifier to predict the miRNAs-drug resistance relationship.

The SVM is an effective classification method and has been widely used in the classification of biological data (43–45). The SVM can transform sample space into high-dimensional or even infinite-dimensional feature space (46). The goal of SVM is to find a hyperplane so that the sample points close to the hyperplane can have a larger distance. The steps of SVM for the algorithm are as follows:

(1) The kernel function *K*(*x_i_*, *x_j_*) and punish parameter *C* need to be selected first. The optimization problem is constructed and solved.


(29) $$\begin{array}{c} \underset{\alpha }{\min}\frac{1}{2}\sum_{i=1}^{N}\sum_{j=1}^{N}\alpha_{i}\alpha_{j}y_{i}y_{j}K(x_{i},x_{j})-\sum_{i=1}^{N}\alpha_{i}\\ \text{ s}.\text{t}.\;\sum_{i=1}^{N}\alpha_{i}y_{i}=0\\ 0\leq \alpha_{i}\leq C,i=1,2,\ldots ,N \end{array}$$

where $K(x_{i},x_{j})=e^{-\frac{ǁx_{i}-x_{j}{ǁ}^{2}}{2\sigma^{2}}}$ . The punish function *C* = 64. The optimal solution is obtained as $\alpha^{*}={\left\{\alpha_{1}^{*},\alpha_{2}^{*},\ldots ,\alpha_{N}^{*}\right\}}^{T}$ (2) A positive component of *a* ^*^ is selected, $0\leq \alpha_{j}^{*}\leq C$ :


(30) $$b^{*}=y_{j}-\sum_{i=1}^{N}\alpha_{i}^{*}y_{i}K(x_{i},x_{j})$$

(3) The decision function is constructed.


(31) $$f(x)=\text{sign }\left(\sum_{i=1}^{N}\alpha_{i}^{*}y_{i}K(x_{i},x_{j})+b^{*}\right)$$

### 2.7 The SVMMDR algorithm

In this section, **Algorithm 1** describes the implementation details of SVMMDR. In lines 2 to 15 of **Algorithm 1**, the low-dimensional inclined diffusion feature matrix *X_M_*_a_nd *X_D_*_a_re obtained by using the inclined random walk with restart and SVD. The hetesim score between any two nodes in a heterogeneous network is obtained from lines 16 to 41. In lines 42 to 45, the combined features is obtained and used to train the SVM classifier. Then the final prediction score is obtained.

Algorithm 1 SVMMDR algorithm.
**Input:** miRNAs set, drugs set, The association matrix of the miRNA-drug resistance, *A*;
**Output:** The gaussian interaction profile kernel similarity matrixs, *GSM* and *GSD*. The miRNAs sequence similarity matrix, *SSM*. The chemical structural similarity matrix, *ESD*. The similarity matrix *SM* and *SD*. Prediction score.
Construct the adjacency matrix *G*;
Obtain the weight matrix *W_MM_
* and *W_DD_*;
Initialize the global transition probability matrix *T_D_* and *T_M_*;
Initialize the transition probability vector for each node 
$P_{0}^{x}(m_{i})=\frac{A(m_{i},d_{x})}{\sum_{n=1}^{nm}A(m_{n},d_{x})}$
, 
$P_{0}^{y}(d_{j})=\frac{A(m_{y},d_{j})}{\sum_{n=1}^{nd}A(m_{y},d_{j})}$

**while** 
$P_{t+1}^{x}-P_{t}^{x}>10^{-10}$
 **do**:
Obtain the updated probability vector:
$P_{t+1}^{y}=(1-r)*T_{D}*P_{t}^{x}+r*P_{0}^{x}$

**end while**

$P_{m*n}=U_{m*m}{\text{Σ}}_{m*n}V_{n*n}^{T}\approx U_{m*d}{\text{Σ}}_{d*d}V_{d*n}^{T}$


$X=U_{n*d}\sum_{d*d}^{1/2}$

Get low-dimensional inclined diffusion feature *X_M_* and *X_D_*
Calculate *I_MD_*(*m_i_*, *d_j_*), *I_DD_*(*d_i_*,*d_j_*),*I_MM_*(*m_i_*, *m_j_*)

${\text{I}}_{\text{MD}}(m_{i},d_{j})=\frac{A(m_{i},d_{j})}{\sum_{k=1}^{nd}A(m_{i},d_{k})}$


${\text{I}}_{\text{DD}}(d_{i},d_{j})=\frac{SD(d_{i},d_{j})}{\sum_{k=1}^{nd}SD(d_{i},d_{k})}$


${\text{I}}_{\text{MM}}(m_{i},m_{j})=\frac{SM(m_{i},m_{j})}{\sum_{k=1}^{nm}SM)(m_{i},m_{k})}$

**for** *l*=1→5 **do**
 Divide the path into two parts.
 **if** *l* % 2 = =0 **then**  
$\rho_{L}={\text{N}}_{1}{\text{N}}_{2}\text{...}{\text{N}}_{\frac{l}{2}+1}$
  
$\rho_{R}={\text{N}}_{\frac{l}{2}+1}{\text{N}}_{\frac{l}{2}+2}\text{...}{\text{N}}_{l+1}$
  
${\text{PM}}_{\rho_{\text{L}}}={\text{I}}_{{\text{N}}_{1}{\text{N}}_{2}}{\text{I}}_{{\text{N}}_{2}{\text{N}}_{3}}\text{...}{\text{I}}_{{\text{N}}_{\frac{l}{2}}{\text{N}}_{\frac{l}{2}+1}}$
  
${\text{PM}}_{\rho_{\text{R}}}={\text{I}}_{{\text{N}}_{\frac{l}{2}+1}{\text{N}}_{\frac{l}{2}+2}}{\text{I}}_{{\text{N}}_{\frac{l}{2}+2}{\text{N}}_{\frac{l}{2}+3}}\text{..}{\text{I}}_{{\text{N}}_{l}{\text{N}}_{l+1}}$

**end if
if** *l* % 2! = 0 **then**  
$\rho_{L_{1}}={\text{N}}_{1}{\text{N}}_{2}\text{..}{\text{N}}_{\frac{l+1}{2}}$
  
$\rho_{L_{2}}={\text{N}}_{1}{\text{N}}_{2}\text{...}{\text{N}}_{\frac{l+3}{2}}$
  
$\rho_{R_{1}}={\text{N}}_{\frac{l+1}{2}}{\text{N}}_{\frac{l+1}{2}+1}\text{...}{\text{N}}_{l+1}$
  
$\rho_{R_{2}}={\text{N}}_{\frac{l+3}{2}}{\text{N}}_{\frac{l+3}{2}+1}\text{...}{\text{N}}_{l+1}$
  
${\text{PM}}_{\rho_{{\text{L}}_{1}}}={\text{I}}_{{\text{N}}_{1}{\text{N}}_{2}}{\text{I}}_{{\text{N}}_{2}{\text{N}}_{3}}\text{...}{\text{I}}_{{\text{N}}_{\frac{l-1}{2}}{\text{N}}_{\frac{l+1}{2}}}$
  
${\text{PM}}_{\rho_{{\text{L}}_{2}}}={\text{I}}_{{\text{N}}_{1}{\text{N}}_{2}}{\text{I}}_{{\text{N}}_{2}{\text{N}}_{3}}\text{...}{\text{I}}_{{\text{N}}_{\frac{l+1}{2}}{\text{N}}_{\frac{l+3}{2}}}$
  
${\text{PM}}_{\rho_{{\text{R}}_{1}}}={\text{I}}_{{\text{N}}_{\frac{l+1}{2}}{\text{N}}_{\frac{l+3}{2}}}{\text{I}}_{{\text{N}}_{\frac{l+3}{2}}{\text{N}}_{\frac{l+5}{2}}}\text{...}{\text{I}}_{{\text{N}}_{l}{\text{N}}_{l+1}}$
  
${\text{PM}}_{\rho_{{\text{R}}_{2}}}={\text{I}}_{{\text{N}}_{\frac{l+3}{2}}{\text{N}}_{\frac{l+5}{2}}}{\text{I}}_{{\text{N}}_{\frac{l+5}{2}}{\text{N}}_{\frac{l+7}{2}}}\text{...}{\text{I}}_{{\text{N}}_{l}{\text{N}}_{l+1}}$
  
$\text{PM}\rho_{L}=\frac{PM_{\rho_{L_{1}}}+PM_{\rho_{L_{2}}}}{2}$
  
$\text{PM}\rho_{R}=\frac{PM_{\rho_{R_{1}}}+PM_{\rho_{R_{2}}}}{2}$

**end if**  
$Hetesim(a,bǀ\rho )=\frac{PM_{\rho_{L}}{\left(PM_{\rho_{R}^{-1}}\right)}^{T}}{ǀǀPM_{\rho_{L}}ǀ{ǀ}_{2}*ǀǀPM_{\rho_{R}^{-1}}ǀ{ǀ}_{2}}$

**end for**
Combined with the inclined diffusion feature and HeteSim score to get the data set
${\text{D}}_{\text{train}}=\left\{\left(x_{1},y_{1}),(x_{2},y_{2}),\ldots ,(x_{N},y_{N}\right)\right\}$


${\text{D}}_{\text{test}}=\left\{\left(x_{1},y_{1}),(x_{2},y_{2}),\ldots ,(x_{N},y_{N}\right)\right\}$

Use D_train_ to train the Support Vector Machines (SVM) as classifier

## 3 Result and discussion

### 3.1 Data sets

The miRNAs-drug resistance association data are downloaded from ncDR database. After deduplication, 85 drugs and 625 miRNAs are obtained, and 2301 miRNAs-drug resistance known association are obtained, all as positive samples. Negative samples are randomly selected from unknown associations with three times the number of positive samples. The final sample dataset is constructed from 2301 positive samples and 6903 negative samples.

### 3.2 Performance measures

The 10-fold Cross-Validation(10-CV) is performed to evaluate the performance of SVMMDR. The process of 10-CV is as follows: the sample data is equally divided into 10 groups. The 9 group of data is used as the training set, and the remaining group is used as the validation set. After ten times of the above process, each of the 10 groups in turn is used as a validation data to obtain 10 performance results. The final performance evaluation is obtained by averaging the 10 performance results. Multiple measures are used to evaluate performance, such as the area under the receiver operating characteristic curves (AUC), recall (REC), accuracy (ACC), F1-score and Matthews Correlation Coefficient (MCC). They can be presented as below:


(32) $$Recall=\frac{TP}{TP+FN,}$$

(33) $$Accuracy=\frac{TP+TN}{TP+TN+FP+FN,}$$

(34) $$F1-Score=\frac{2\times TP}{2TP+FP+FN,}$$

(35) $$MCC=\frac{TP\times TN-FP\times FN}{\sqrt{\left(TP+FP)(TP+FN)(TN+FP)(TN+FN\right)}}$$

where the *TP* is the number of samples that are correctly classified as positive, the *FP* is the number of samples that are misclassified as positive, the *TN* represents the number of samples that are correctly classified as negative, and the *FN* is the number of samples that are misclassified as negative.

### 3.3 Performance comparison with existing machine learning methods

In order to reflect the performance of SVM, the proposed SVMMDR methods will be compared with the following solution, including using logistic regression (LR) as a classifier, the use of random forests (RF) used as a classifier, K nearest neighbor (KNN) as a classifier. The same features of the same training sample are used to train the corresponding classifiers. To get performance, the 10-flod cross-validation is applied. For KNN classifier, the 10 nearest neighbors and leaf size of 20 point is used. The RF builds a number of decision tree classifiers trained on a set of randomly selected samples of the benchmark to improve the performance. For LR, the maxiter and tol parameters are optimized to 500 and 0.001, respectively.

**Figure 2:**
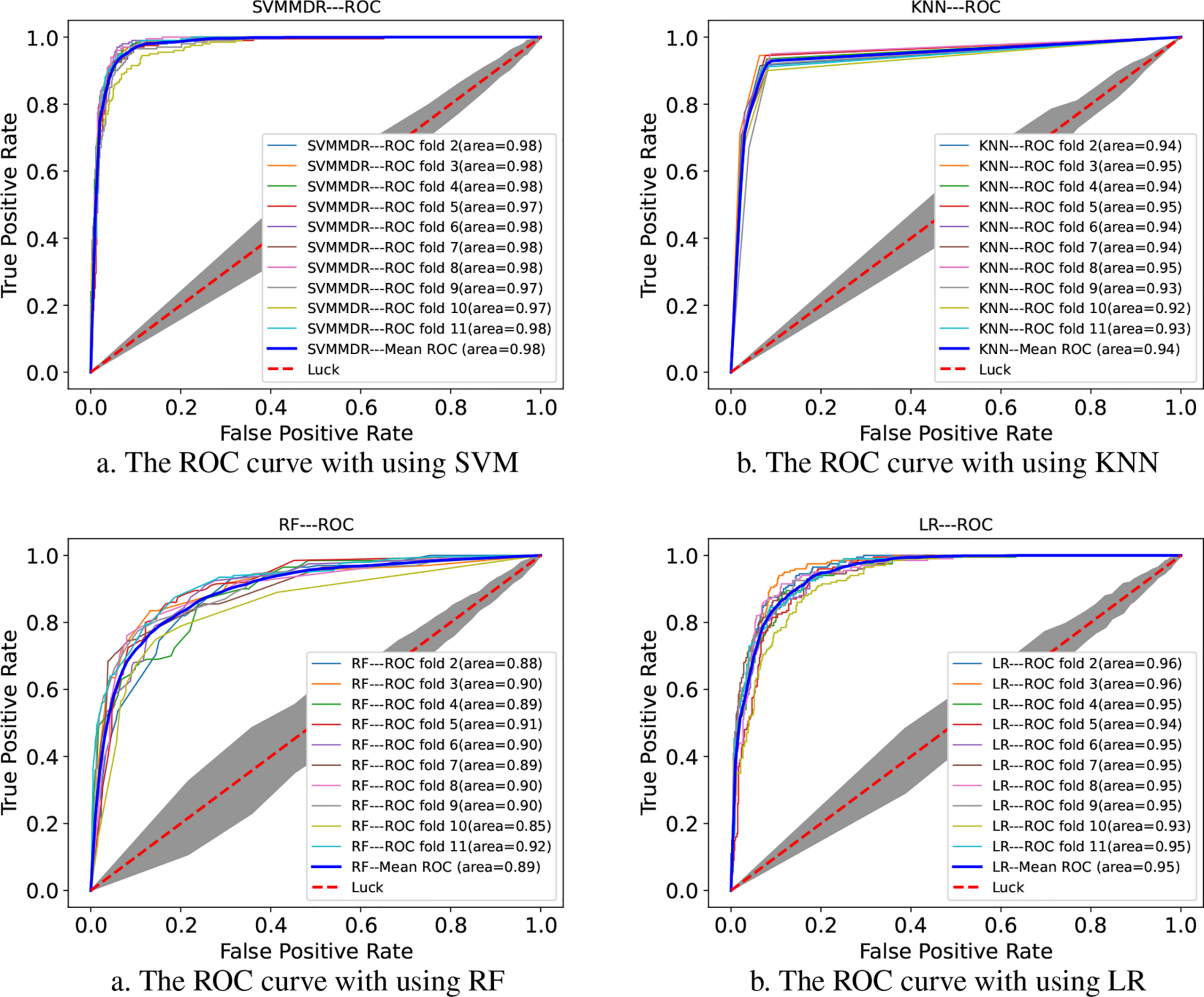
The ROC curve comparison with existing machine learning methods.

**Figure 2** indicates the ROC curves of SVMMDR using other classifiers. The AUC of SVMMDR, KNN, RF and LR are 0.978, 0.939, 0.892 and 0.948. Furthermore, **Table 2** shows the values of performance measures such as ACC, Pre, Recall, F1-score, MCC. The results show that the AUC value obtained by SVMMDR is the highest. The value of performance measure is also better than other classifiers. The SVM classifier can achieve effective classification by mapping features to higher dimensional space through kernel function changes. At the same time, the optimal solution is obtained with constraints, which can make the classification more accurate.

**Table 2:** Performance comparison of existing machine learning methods.

Method	ACC	Pre	RECALL	F1-score	MCC
SVMMDR	0.9393	0.8705	0.8905	0.8800	0.8399
RF	0.8381	0.81733	0.4625	0.5704	0.5236
LR	0.8925	0.8020	0.7565	0.7785	0.7082
KNN	0.9080	0.8936	0.7175	0.7957	0.7448

### 3.4 Performance comparison with different topological features

**Figure 3:**
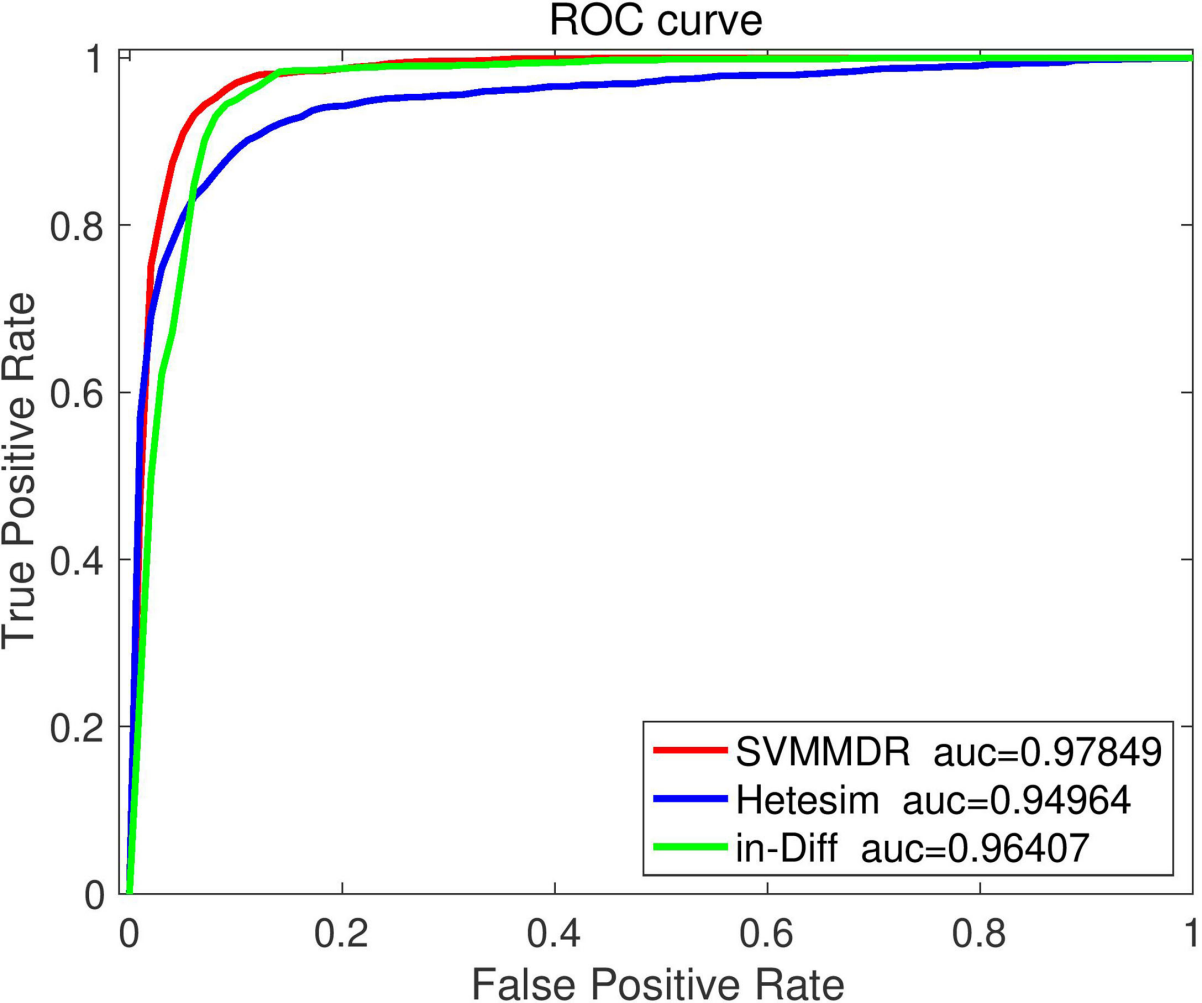
The ROC curve comparison with different feature.

**Figure 4:**
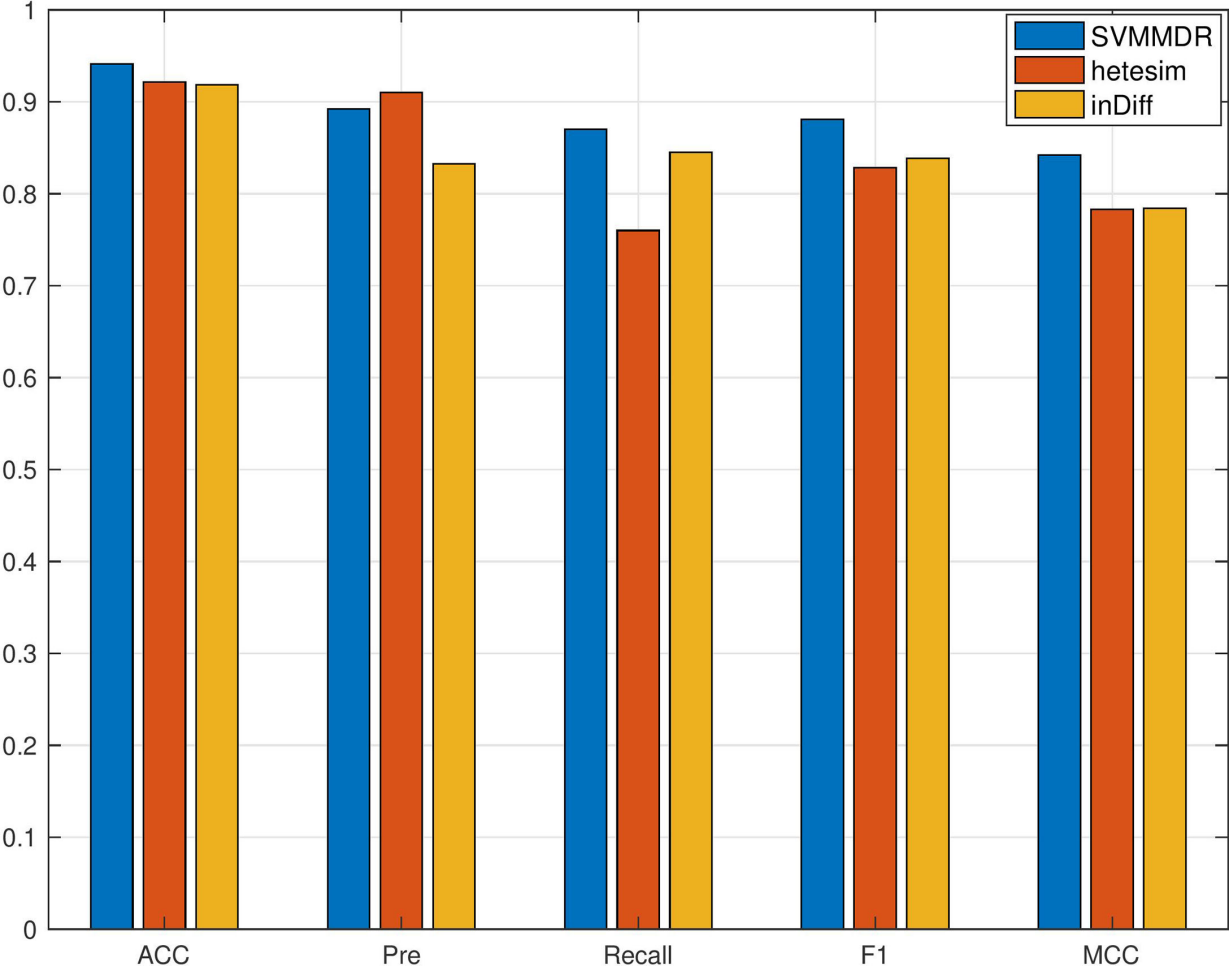
The performance comparison with different feature.

To demonstrate the advantages of combining features in SVMMDR, different feature groups (hetesim+ inclined diffusion feature, hetesim feature, and inclined diffusion feature) are used for comparison experiment. The comparison results are shown in **Figures 3** and **4**. Denote “SVMMDR”, “Hetesim” and “in-Diff” as the combination feature, hetesim feature and inclined diffusion feature. **Figure 3** shows the ROC curves of different feature groups. It can be seen that the combination of hetesim and inclined diffusion obtained a higher AUC than the two separate feature, and the AUC obtained by inclined diffusion feature alone is higher than that obtained by hetesim alone. **Figure 4** represents the performance achieved for the different feature groups. It can also see that the combination of the two features has best performance. Although the AUC of inclined diffusion feature reaches 0.96, the Pre, F1 and MCC are all relatively low. The combination of inclined diffusion feature and hetesim feature can solve this problem and improve performance.

### 3.5 Performance comparison with existing methods

To further illustrate the superiority of the proposed method, the SVMMDR is compared with existing miRNA-resistance prediction algorithms, such as GCMDR, MDIPA and BiRW-MD, all of which use the sample set in this paper. Performance measures are obtained by performing 10-fold cross-validation.

GCMDR (35): Data from multiple data sources are fused. The latent factor method are constructed using graph convolution to learn the graph embedding feature of miRNAs and drugs, and end-to-end prediction method are built.

MDIPA (37): The identification of potential miRNAs-drug interactions is seen as a matrix completion problem, the unknown associations are predicted based on weighted non-negative matrix factorization. The path-based miRNAs similarity matrix and drugs similarity matrix based on drugs structure information are obtained, which are combined with extracted drugs and miRNAs neighbor information for prediction.

BiRW-WD (33): The multiple similarity networks and miRNAs-drugs association network are integrated to construct a heterogeneous network. In the heterogeneous networks, the bi-directional random walk (BiRW) are used to predict potential miRNAs-drug effect associations.

**Figure 5:**
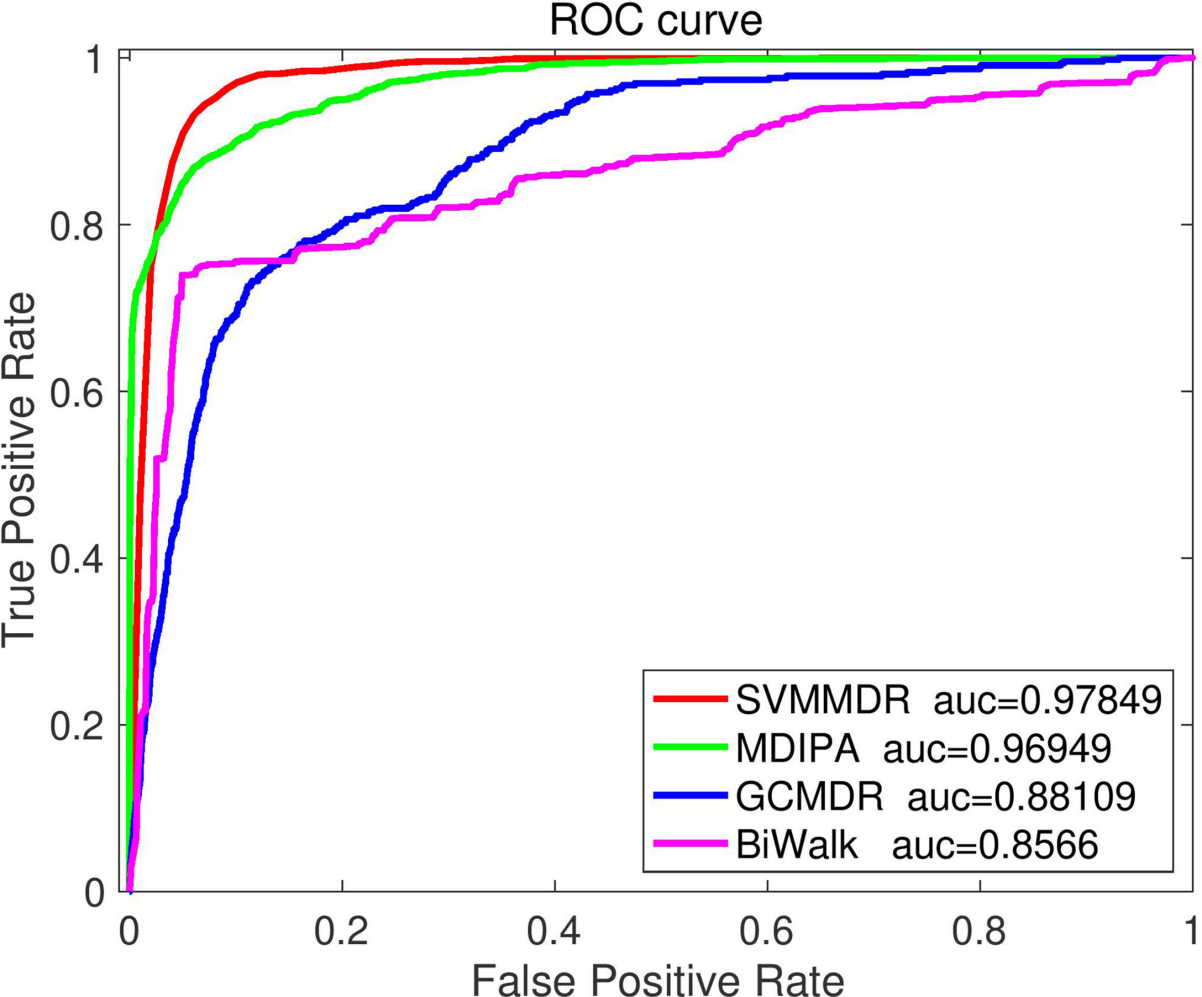
The ROC curve comparison with existing methods.

**Figure 5** illustrates the comparison results. It can be seen that the proposed SVMMDR method achieves the best performance. The reasons are as follows: (1) The drug group and miRNA group are introduced. When restart random walk is used to obtain diffusion feature, the walker is more inclined to select the node of the next walk. The inclined diffusion feature contribute to the prediction accuracy. (2) The hetesim score is obtained from the path information of two nodes in the heterogeneous network. Regardless of the same or different node types, the hetesim measures their correlation within a unified framework. At the same time, according to the search path between two nodes, the measure between node pairs is defined by following a sequence. (3) The SVM with high accuracy is used as the classifier.

### 3.6 Case study

In order to illustrate the effectiveness of the proposed method, we present a case study of the drug 5-fluorouracil(5-FU). The 5-FU is an antimetabolite drug widely used in cancer treatment, especially colorectal cancer (CRC)Longley et al. (47). There are 244 miRNAs related to 5-FlU in ncDR database. We remove these associations in the association matrix A and use the rest as test data. The SVMMDR algorithm proposed in this paper is used for prediction, and 174 miRNAs with prediction scores greater than 0.95 are obtained. For the top 20 predicted miRNAs, we verify whether predicted miRNAs-drug resistance associations are confirmed by searching the PubMed literature. **Table 3** indicates the miRNAs and the PMIDs of publications mentioning the association between miRNAs and 5-FU. For example, miR-21 expression levels are confirmed to lead to 5-Fluorouracil resistance Tomimaru et al. (48). The miR-23a enhances 5-FU resistance in microsatellite instability (MSI) CRC cells through targeting ABCF1 Li et al. (49).

**Table 3:** The top 20 predicted miRNA related to 5-FU.

miRNA	PMID	miRNA	PMID
miR-3665	25117811	let-7b	22382630
miR-3619	25117811	miR-99a	24304648
miR-3141	25117811	miR-4465	25117811
miR-1234	25117811	miR-4484	25117811
miR-1275	25356050	miR-887	25117811
miR-29b-2	23167930	miR-4750	25117811
miR-423	25117811	miR-221	25117811
miR-107	22382630	miR-191	24304648
miR-296	20485139	miR-23a	24249161
miR-483	24304648	miR-21	24275137

## 4 Conclusion

More and more evidence indicates that miRNA expression level is related to drug resistance, affecting the therapeutic effect of disease. Predicting the association between miRNA-drug resistance can help to select more appropriate drugs for clinical treatment and promote the cure of disease. However, there are also very few computation-based predictive tools for miRNA- drug resistance.

Therefore, in this paper, a method based on the Support Vector Machines to predict the relationship between MiRNA and Drug Resistance (SVMMDR) is proposed. The SVMMDR integrates miRNAs-drug resistance association, miRNAs sequence similarity, drug chemical structure similarity and other similarities, extracts path-based hetesim features, and obtains inclined diffusion features through inclined restart random walk. The machine learning algorithm SVM is used to predict the association between miRNAs and drug resistance.

The 10-fold cross-validation is used to assess the performance of SVMMDR. The area under the ROC curve AUC is used as a measure of performance. The AUC of SVMMDR reaches 0.978. The results show that SVMMDR has a significant performance advantage.

## Data availability statement

Publicly available datasets were analyzed in this study. This data can be found here: http://www.jianglab.cn/ncDR/.

## Author contributions

TD, ZFK, and LD conceived this work and designed the experiments. TD and ZFK carried out the experiments. TD and ZFK collected the data and analyzed the results. TD, ZFK and LD wrote, revised, and approved the manuscript. All authors contributed to the article and approved the submitted version.

## Funding

This work was supported in part by the National Natural Science Foundation of China under Grants Nos. 62072477, 61309027, 61702562 and 61702561, the Hunan Provincial Natural Science Foundation of China under Grants No.2018JJ3888, the Scientific Research Fund of Hunan Provincial Education Department under Grant No.18B197, the National Key R&D Program of China under Grant No.2018YFB1700200, the Open Research Project of Key Laboratory of Intelligent Information Perception and Processing Technology (Hunan Province) under Grant No.2017KF01, the Hunan Key Laboratory of Intelligent Logistics Technology 2019TP1015.

## Acknowledgments

We would like to thank the Experimental Center of School of Computer and Information Engineering, Central South University of Forestry and Technology, for providing computing resources.

## Conflict of interest

The authors declare that the research was conducted in the absence of any commercial or financial relationships that could be construed as a potential conflict of interest.

## Publisher’s note

All claims expressed in this article are solely those of the authors and do not necessarily represent those of their affiliated organizations, or those of the publisher, the editors and the reviewers. Any product that may be evaluated in this article, or claim that may be made by its manufacturer, is not guaranteed or endorsed by the publisher.
